# Dental Clinic Architecture Prevents COVID-19-Like Infectious Diseases

**DOI:** 10.1177/1937586720943992

**Published:** 2020-07-29

**Authors:** Samia Naz Isha, Ashfi Ahmad, Russell Kabir, Ehsanul Hoque Apu

**Affiliations:** Research Associate, Department of Public Health Sciences, 535595 Centre for Injury Prevention and Research (CIPRB), Dhaka, Bangladesh; Part-time Lecturer, Department of Architecture, North South University, Dhaka, Bangladesh; Senior Lecturer in Research Methods, School of Allied Health, Faculty of Health, Education, Medicine and Social Care, 2369 Anglia Ruskin University, Chelmsford, UK; Postdoctoral Research Associate and Faculty, Department of Biomedical Engineering, 3078 Michigan State University, East Lansing, MI, USA

Dental clinics are at high risk of the coronavirus disease (COVID-19) and cross-infection due to the use of instruments that produce aerosols, droplets of secretions, saliva, and blood. These can cause transmission between dental practitioners and patients. Large droplets could potentially cause the viral transmission to subjects nearby, whereas smaller droplets can be suspended in the air for some time. Other than spreading from an infected person, there is a risk of being exposed to asymptomatic undiagnosed patients. It is essential for a dental clinic to ensure hand-hygiene, personal-protective equipment, and caution in aerosol-generating procedures (Sabino-Silva et al., 2020); the design or layout plays an important role in infection prevention and cross-transmissions (Krishnan & Pandian, 2016). There are two approaches to prevent cross-contamination, prevention from patients to doctors (vice-versa), and disinfect surfaces and objects (Palenik et al., 2000).

In any dental clinic, it is imperative to separate two areas: treatment and nontreatment zones. Patients are in direct contact in the treatment zone, where different procedures take place, including impression taking and radiography. Nontreatment zones include waiting areas, reception, lavatory, breakrooms, and office space (Krishnan & Pandian, 2016). The treatment zone should be spacious enough for free movement and efficient functioning to avoid exposed surfaces being contaminated with aerosols. The reception areas, waiting room, and consultation rooms should be near to the entrance. Short and quick treatment units should be placed adjacent to the consultation rooms with longer treatment units at the end of the space. Laboratories and radiograph options should always be near to treatment areas for easy access (Krishnan & Pandian, 2016).

The COVID-19 is caused by SARS-CoV-2 virus, and depending on materials, it remains active on inanimate objects from 2 hours to 9 days (Ren et al., 2020). Aerosols/droplets produced during dental procedures may remain in the air for 3 hours (Meselson, 2020). Surface disinfectants can be used to clean the surface as primary protection from the spread of infection. Certain metals carry antimicrobial properties preventing surfaces from being a reservoir for microbes. Metals containing copper have antiviral properties, and its alloys, like brass and bronze, are considered antimicrobial. It takes 40 min on a brass surface and 2 hours on a copper (70%) nickel surface to inactivate the SARS-CoV-2 (Ren et al., 2020).

It may be recommended that copper “should” be used for frequently touched surfaces like door handles, countertops, and interior cladding material. Copper floor, paint, roof, or wall installation might purify surrounding air, continuously reducing dependence on an air purifier. The use of air conditioners in warm climate is mandatory, where copper alloy air vents or air conditioning ducts might help purify circulating air while cooling it. Sinks and faucets should be designed for minimal hand contact. Foot pedal or arm-operated faucets should be introduced with auto-dispensing soap stations, and paper towels should be used instead of cloth towels (Krishnan & Pandian, 2016). To avoid cross-transmission, waste management should include these steps: segregation, decontamination, deformation containment, transportation, and final disposal. Medical waste types, collection bins and boxes, should be labeled accordingly, where infectious waste must be separated from sharp, toxic, hazardous, and stationary waste (Krishnan & Pandian, 2016).

***It may be recommended that copper “should” be used for frequently touched surfaces like door handles, countertops, and interior cladding material. Copper floor, paint, roof, or wall installation might purify surrounding air, continuously reducing dependence on an air purifier***

Samia Naz Isha, BDS (Dentistry), MPH (Public Health), MSc (International Health)*Research Associate, Department of Public Health Sciences, Centre for Injury
Prevention and Research (CIPRB), Dhaka, Bangladesh*Ashfi Ahmad, BSc (Architecture), MSc (Eco-cities)*Part-time Lecturer, Department of Architecture, North South University, Dhaka,
Bangladesh*Russell Kabir, BDS (Dentistry), MSc (Public Health), MSc (Research Methods), PhD (Public
Health)*Senior Lecturer in Research Methods, School of Allied Health, Faculty of Health,
Education, Medicine and Social Care, Anglia Ruskin University, Chelmsford, UK*Ehsanul Hoque Apu, BDS (Dentistry), MSc (Oral Pathology), PhD (Cancer Medicine)*Postdoctoral Research Associate and Faculty, Department of Biomedical Engineering,
Michigan State University, East Lansing, MI, USA*
